# A frequent SNP in TRIM5α strongly enhances the innate immune response against LINE-1 elements

**DOI:** 10.3389/fimmu.2023.1168589

**Published:** 2023-04-26

**Authors:** Justine Lagisquet, Marcus Conrad, Sabine Wittmann, Bianca Volkmann, Hannah Weissinger, Heinrich Sticht, Thomas Gramberg

**Affiliations:** ^1^ Institute of Clinical and Molecular Virology, Friedrich-Alexander-Universität Erlangen-Nürnberg, Erlangen, Germany; ^2^ Division of Bioinformatics, Institute of Biochemistry, Friedrich-Alexander-Universität Erlangen-Nürnberg, Erlangen, Germany

**Keywords:** TRIM5α, LINE-1, retroelements, NF-κB, AP-1, restriction factor, pattern recognition receptor, H43Y

## Abstract

The intracellular restriction factor TRIM5α inhibits endogenous LINE-1 retroelements. It induces innate immune signaling cascades upon sensing of cytoplasmic LINE-1 complexes, thereby underlining its importance for protecting the human genome from harmful retrotransposition events. Here, we show that a frequent SNP within the RING domain of TRIM5α, resulting in the variant H43Y, blocks LINE-1 retrotransposition with higher efficiency compared to TRIM5α WT. Upon sensing of LINE-1 complexes in the cytoplasm, TRIM5α H43Y activates both NF-κB and AP-1 signaling pathways more potently than TRIM5α WT, triggering a strong block of the LINE-1 promoter. Interestingly, the H43Y allele lost its antiviral function suggesting that its enhanced activity against endogenous LINE-1 elements is the driving force behind its maintenance within the population. Thus, our study suggests that the H43Y variant of the restriction factor and sensor TRIM5α persists within the human population since it preserves our genome from uncontrolled LINE-1 retrotransposition with higher efficiency.

## Introduction

TRIM5α is a member of the TRIM protein family and blocks retroviral infection at an early post-entry step by targeting incoming viral cores (1). It contains the characteristic amino-terminal tripartite motif (TRIM) and a unique carboxy-terminal PRY-SPRY or B30.2 (SPRY) domain that is responsible for direct interaction with retroviral cores. The TRIM motif includes a RING domain, which comprises an E3 ubiquitin ligase activity and mediates innate immune signaling upon binding to retroviral cores (2–4). TRIM5α has been suggested to act as pattern recognition receptor (PRR) detecting incoming retroviral cores and initiating NF-κB and AP-1 signaling upon binding. While human TRIM5α efficiently restricts the infectivity of several retroviruses, such as N-tropic murine leukemia virus (N-MLV), it only weakly inhibits HIV-1 replication, in contrast to various simian TRIM5α orthologues. The decreased ability to restrict HIV-1 has been pinpointed to a weak interaction of the SPRY domain with HIV-1 cores (5–9). However, recent literature suggest a more complex role for human TRIM5α in restricting HIV-1 replication. In response to type I IFN, human TRIM5α has been reported to inhibit HIV-1 through induction of the immunoproteasome (10). In addition, it has been found that, in contrast to HIV-1 isolates of the pandemic group M, non-pandemic HIV-1 group O or HIV-2 viruses are indeed restricted by human TRIM5α (11). In line with its proposed role in direct pathogen interaction, strong evidence for an ancient positive selection process in the TRIM5α gene, predating the occurrence of primate lentiviruses such as HIV-1, has been described (12, 13). This evolutionary process has been suggested to be driven by confrontations with exogenous and endogenous retroviruses, most likely γ-retroviruses which have episodically invaded human genomes (12). Multiple TRIM5α alleles within the human population have been identified and the antiretroviral activities of the most frequent single nucleotide polymorphisms (SNPs) in TRIM5α have been determined (14). One of the most common SNPs in TRIM5α results in the amino acid substitution histidine to tyrosine at position 43 (H43Y) and is located within the RING domain of TRIM5α (15). According to the Genome Aggregation Database (gnomAD), the overall allele frequency of the variant rs3740996, which translates to 43Y, is 0.12, resulting in homozygosity rate of 1.29% (16). Interestingly, the geographic distribution of the allele varies from the highest frequencies found in Amish (0.17) and East Asian (0.16) samples to really low frequencies in samples from African descent (0.06). Conflicting results exist whether the H43Y allele positively or negatively affects HIV infectivity, replication, or pathogenicity *in vivo* (17–21), most likely due to differences in study design. Nevertheless, several studies clearly describe a reduced antiviral activity of TRIM5α H43Y compared to WT protein in cell culture models (14, 22). Thus, the question arises why the TRIM5α allele H43Y is present within the human population at relative high numbers despite its loss in antiviral activity.

Recently, we were able to extend the role of human TRIM5α as innate receptor and restriction factor and found that it also blocks and senses the replication of long interspersed element 1 (LINE-1) (23). LINE-1 retroelements are the only autonomously active mobile DNA elements present in humans and their sequences comprise ~17% of the human genome (24). Although most sequences are degenerated, about 100 full-length and potentially replication-competent copies still exist (25). Following LINE-1 transcription and translation, the two encoded proteins ORF1p and ORF2p bind “in cis” to the RNA, from which they just have been translated, to form ribonucleoprotein complexes (RNP) in the cytoplasm (26, 27). These complexes are then relocated into the nucleus where the enzymatically active ORF2p cleaves genomic DNA at a new locus within a specific T/A-rich target site and reverse transcribes LINE-1 RNA directly into the host genome through a process called target-primed reverse transcription (TPRT). Previously, we found that TRIM5α interferes with the LINE-1 replication cycle at different steps (23). First, TRIM5α binds LINE-1 RNPs in the cytoplasm and potentially directly inhibits LINE-1 in the cytoplasm. Secondly, upon binding to RNPs TRIM5α initiates innate immune signaling pathways followed by downregulation of the LINE-1 promoter activity (23). Thus, our findings suggests that human TRIM5α protects the integrity of our genome by sensing and downregulating excess LINE-1 activity. Interestingly, Sawyer and colleagues reported that a burst of positive selection in the TRIM5α gene predates the occurrence of primate lentiviruses (12), making endogenous mobile genomic elements a potential candidate for the selective pressure on TRIM5α. Thus, we asked whether the high prevalence of the H43Y allele might be connected to the activity of TRIM5α against LINE-1.

Here, we report that the H43Y variant of TRIM5α blocks LINE-1 retrotransposons more efficiently than the WT protein. H43Y also activated both NF-κB and AP-1 signaling pathways more potently than the WT protein suggesting that the enhanced sensitivity of the receptor H43Y is coupled to the amino acid substitution within the E3-ubiquitin ligase domain. We tested various known SNPs for their capacity to inhibit LINE-1 retroelements. Interestingly, we found that the common variant H43Y showed enhanced anti-LINE-1 activity compared to WT protein, suggesting that this property might be the driving force behind its maintenance within the human genome.

## Materials and methods

### Cell lines

HEK293T cells (293T), feline kidney cell line CRFK, and the retroviral packaging cell line GP2-293 were maintained in Dulbecco´s Modified Eagle Medium (DMEM) supplemented with 10% fetal bovine serum, 100 U/ml penicillin, 10 µg/ml streptomycin and 1 mM glutamine. 293T expressing shRNA targeting TRIM5α (293T- shTRIM5α) were generated by lentiviral transduction as described previously (23). Cells were then selected and further cultured in complete DMEM supplemented with 2.5 µg/ml puromycin. CRFK were stably transduced with retroviral vectors expressing either an empty vector or the different variants of TRIM5α and were selected with 1 mg/ml G418 two days post-infection.

### Plasmids

Plasmids encoding N-terminal HA-tagged human and rhesus TRIM5α (allele *Mamu3*) were a kind gift of Andrea Kirmaier (Harvard Medical School and Boston College, Massachusetts). Human and rhesus TRIM5α sequences were inserted into the retroviral expression vector pQCXIN *via* the AgeI and EcoRI restriction sites, resulting in pQCXIN-HA-huTRIM5α. Human TRIM5α single nucleotide polymorphism (SNP)-containing variants were generated by site-directed mutagenesis followed by overlapping PCR and inserted into pQCXIN *via* AgeI and EcoRI. ShRNA targeting TRIM5α (GGTTAGAGGAAGGAGTTAAAT) was cloned into the lentiviral vector pLKO.1-puro *via* AgeI and EcoRI as described previously (23). ShRNA-resistant TRIM5α variants (WT^R^, H43Y^R^, R437C^R^) were generated by overlapping PCR mutagenesis. The LINE-1-GFP retrotransposition-competent reporter construct 99 PUR RPS EGFP as well as the defective construct 99 PUR JM111 EGFP (JM111) were a kind gift of John Goodier (John Hopkins University) (28). Full length LINE-1 under the control of the CMV promoter (pAD2TE1) has been described previously (29). The LINE-1-luc reporter construct has been generated by amplification of the promoter sequence of 99 PUR RPS EGFP and inserted into the pGL3 Basic vector (Promega). The plasmid expressing the luciferase gene under the control of AP-1 binding sites was a kind gift of Reinhard Voll (Universitätsklinikum, Freiburg). NF-κB luciferase reporter construct was purchased from Stratagene (#219078). pMD2.G (VSV-G) was a gift from Didier Trono (Addgene plasmid #12259) and pCIG3 N (N-tropic MLV gag-pol) was a kind gift from Jeremy Luban (Addgene plasmid #132941) (30). The envelope-deficient HIV-1 GFP reporter construct pNL43-ΔE-CMV-EGFP has been described previously (31).

### Lentiviral and retroviral production

To generate VSV-G pseudotyped HIV-GFP reporter virus, 293T cells were transfected with pNL43-ΔE-CMV-EGFP and pMD2.G at a mass ratio of 4:1. N-MLV reporter virus was generated by transfecting pMX-GFP, pCIG3 N, and pMD2.G into 293T cells at a mass ratio of 2:2:1 by calcium phosphate precipitation. Lentiviral particles expressing shRNA targeting TRIM5α were generated by cotransfection of pLKO.1-shTRIM5α, the packaging plasmid pCMVΔR8.91, and pMD2.G into 293T cells. For retroviral vectors used for to generate stable CRFK cells lines, pQCXIN plasmids encoding the different TRIM5α variants were cotransfected with pVSV-G into the packaging cell line GP2-293 at a mass ratio of 2:1. Supernatants were collected 48 h posttransfection, cells debris was removed by centrifugation and filtration through 0.45 µm membranes (Sartorius). The N-MLV-GFP reporter virus was concentrated using size exclusion filters according to the manufacturer’s instructions (Amicon 100, Merck Millipore).

### Lentiviral and retroviral infections

For lentiviral and retroviral infections, 1x10^5^ CRFK cells expressing the different variants of TRIM5α were infected in a 12-well plate with the different GFP reporter viruses at the indicated multiplicity of infection (MOI). Three days postinfection, cells were harvested, fixed in 2% PFA, and analyzed by flow cytometry.

### LINE-1 retrotransposition assay

To analyze LINE-1 retrotransposition in cell culture, 293T or 293T-shTRIM5α cells were transfected with the LINE-1-GFP reporter construct 99 PUR RPS EGFP together with either an empty vector or the plasmids encoding the variants of TRIM5α in a ratio 3:1 using calcium phosphate coprecipitation. After two days, 2.5 µg/ml puromycin was added to the medium for three additional days. Five days posttransfection, cells were harvested and GFP-positive cells were quantified by flow cytometry as a surrogate for successful LINE-1 retrotransposition. The retrotransposition-defective plasmid 99 PUR JM111 EGFP was used as a negative control. To assess the impact of TRIM5α H43Y-dependent signaling on LINE-1 retrotransposition, 300 nM of the TAK1 inhibitor 5Z-7-oxozeaenol (Calbiochem, 499610) or a combination of 1 µM of IKK inhibitor VII and 5 µM of IKK inhibitor XII (Calbiochem, 401486 and 401491) was added to the medium 6 h and 48 h posttransfection.

### Droplet digital PCR

293T-shTRIM5α were cotransfected with the LINE-1-GFP reporter construct and the shRNA-resistant TRIM5α variants. Five days posttransfection, genomic DNA was extracted using QIAamp DNA Blood Mini Kit (Qiagen) according to the manufacturer´s instructions. DNA concentration was measured by spectrophotometry (NanoDrop Lite, Thermo Scientific). PCR reactions were performed in 96-well plates with 100 ng of genomic DNA combined with 2x ddPCR Supermix (no dUTP, BioRad), 900 nM 5´oligo targeting EGFP (5´- TGTTCTGCTGGTAG-3’), 900 nM 3´oligo targeting EGFP (5´-GGCATCAAGGTGAAC-3´) and 250 nM of a FAM/BHQ1-labeled probe spanning the splice junction of the LINE-1-GFP reporter construct (FAM-Tcggccagctgcac-BHQ1). Normalization of LINE-1 GFP copy numbers to host genome was done by adding ribonuclease P subunit p30 (RPP30) gene-specific primers and a HEX-labeled probe (BioRad). Droplets were generated through combination of the PCR reaction and lipid oil using a QX200 Droplet Generator (BioRad). PCR was run on a BioRad T100 Thermal cycler and fluorescent signals were measured and analyzed using a BioRad Plate Reader and Quanta Soft Analysis Pro Software respectively.

### Immunoblotting

Cells were lysed in NP-40 lysis buffer (10 mM Tris-HCl pH 7.5, 150 mM NaCl, 2 mM EDTA, 0.5% NP-40, and freshly supplemented with Halt Protease Inhibitor). Protein lysates were quantified by Bradford assay (Carl Roth). 10-30 µg of lysate were separated by SDS-PAGE, transferred onto Immobilon-P PVDF membrane (Merck-Millipore), and probed with an anti-HA antibody (3F10, Sigma-Aldrich). For loading controls, membranes were probed with anti-HSP90 α/β antibody (Santa-Cruz). Membranes were probed with anti-rat or anti-mouse horseradish peroxidase (HRP)-labelled secondary antibodies (Thermo Fischer Scientific; Cell Signaling respectively) and signals were visualized using an INTAS Advanced Fluorescence Imager (INTAS).

### Luciferase reporter assays

3x10^4^ 293T-shTRIM5α cells were seeded in 96-well plates and transfected with 100 ng of L1 5’UTR-luc together with an empty vector or increasing amounts of shRNA-resistant TRIM5α WT or H43Y using Lipofectamine2000 (Life Technologies). To assess the impact of LINE-1 RNPs on the TRIM5α H43Y-dependent inhibition, increasing amounts of LINE-1 expressing plasmid pAD2TE1 were co-transfected with 25 ng of either an empty vector or TRIM5α WT^R^ or H43Y^R^. For AP-1 and NF-κB signaling assays, cells were transfected with either 10 ng of AP-1-luc or 2.5 ng of NF-κB-luc and increasing amounts of TRIM5α WT or H43Y in presence or absence of the pAD2TE1 LINE-1 construct. In assays using small molecule inhibitors, cells were treated 6 h posttransfection with either 300 nM 5Z-7-oxozeaenol (Oxo) or a combination of 1 µM of IKK inhibitor VII and 5µM of IKK inhibitor XII (IKKi). Two days posttransfection, cells were lysed with 5x Cell Culture Lysis reagent (Promega) and luciferase activity was measured using Luciferase Assay System (Promega) on a Berthold microplate reader.

### Structure prediction and energy calculations

Energetic analyses were performed using the PositionScan algorithm of the program Fold-X (version 5) (32). To evaluate the effects of the mutation to tyrosine, the ΔG value of the folding free energy between WT (H43) and Y43 was calculated. The analysis was done for all 20 conformers of the TRIM5α-Ensemble determined by NMR-spectroscopy (PDB entry 2ECV). The effect of the H➔Y exchange was separately analyzed for models containing either neutral or charged histidine, which were created by the BUILDMODEL routine of Fold-X.

### Cell viability assay

1.5 x 10^5^ 293T-shTRIM5α cells were treated in a 12-well plate with 300 nM of 5Z-7Oxozeaenol (Oxo) or a combination of 1 µM of IKKi VII and 5µM IKKi XII. Two days after, the medium was removed and replaced with fresh medium containing the corresponding doses of inhibitors for three additional days. Five days after the first treatment, cells were stained with eBioscience™ Fixable Viability Dye eFluor™ 780 (Invitrogen) and cell viability was measured by flow cytometry.

## Results

### TRIM5α H43Y efficiently blocks LINE-1 retrotransposition

**Figure 1:**
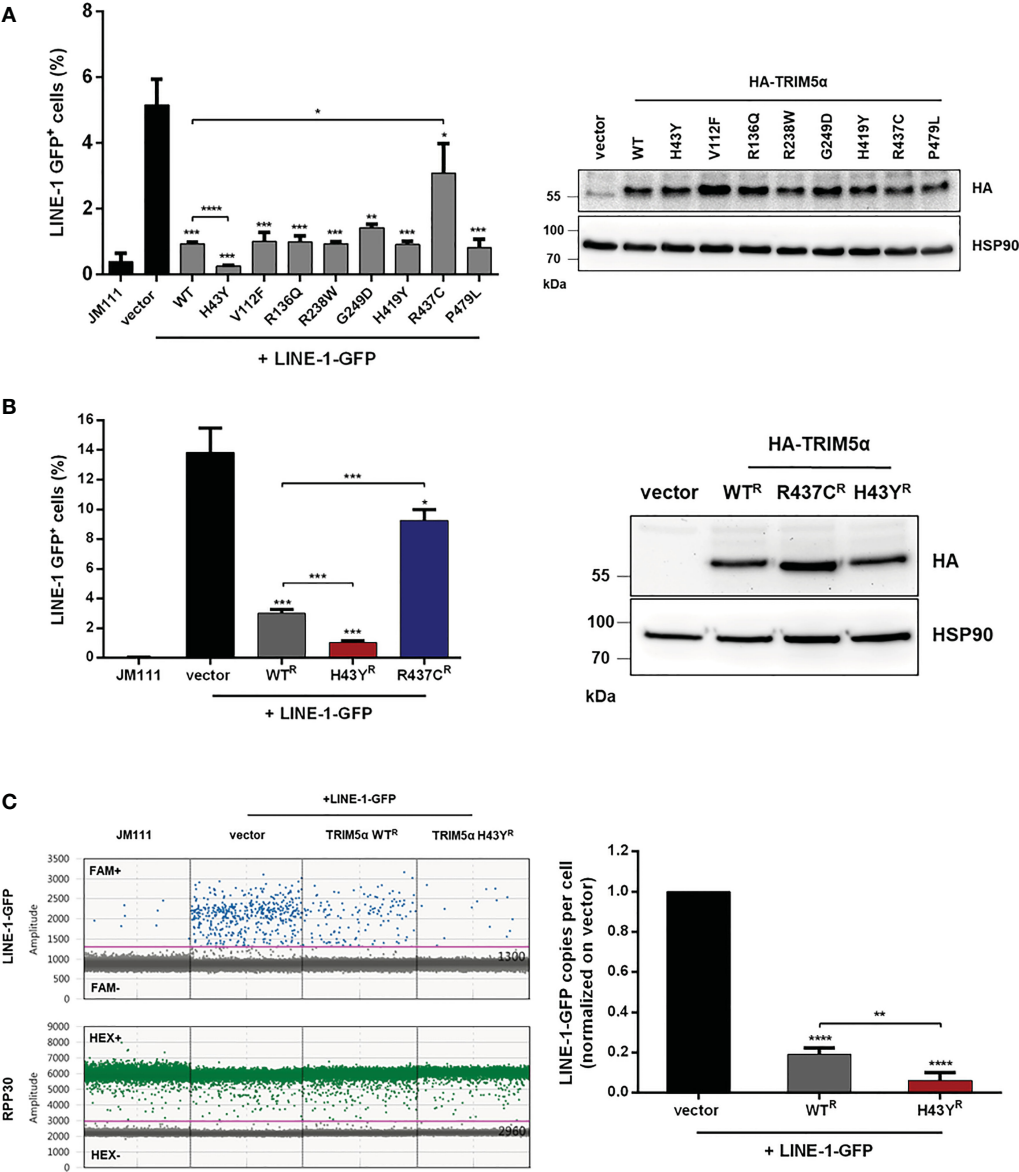
TRIM5α H43Y efficiently blocks LINE-1 retrotransposition. **(A)** 293T or **(B)** 293T-shTRIM5α were transfected with LINE-1-GFP reporter plasmid or a retrotransposition-defective construct (JM111) together with empty vector or vector expressing the indicated single nucleotide polymorphism in TRIM5α. Expression of the HA-tagged TRIM5α constructs was confirmed by immunoblot using an HA-specific antibody. Five days posttransfection, GFP-positive cells were quantified by flow cytometry. The percentage of GFP-positive cells is presented as mean of triplicate transfections. Error bars represent SD. **(C)** 293T-shTRIM5α cells were transfected with LINE-1-GFP and the indicated TRIM5α variants. Five days posttransfection, genomic DNA was extracted and LINE-1 integration events were quantified by droplet digital PCR (ddPCR) using oligos targeting the spliced GFP reporter and normalized on the housekeeping gene RPP30 (copies per cell). Events in vector control cells are set to 100% (1.0). Results are shown as mean of quadruplicate transfections with error bars representing SD. One out of three independent experiments is shown. Statistical analysis were done using two-tailed, unpaired t-test. *P<0.1, **P<0.01, ***P<0.001, ****P<0.0001. ^R^ shRNA-resistant TRIM5α WT, H43Y, or R437C.

Several nonsynonymous SNPs in human TRIM5α have been identified and the most common alleles have been tested for their ability to restrict retroviral replication *in vitro* (14). While the antiviral activity of most alleles is not affected, the TRIM5α polymorphism H43Y showed a severe loss in retroviral restriction. Previously we found that TRIM5α also senses and restricts endogenous LINE-1 retroelements (23). To assess whether the variants of TRIM5α differ in their activity against LINE-1, we tested the most frequent alleles present in the human population, carrying the SNPs H43Y, V112F, R136Q, R238W, G249D, H419Y, or P479L (15), for their anti-LINE-1 activity in LINE-1 reporter assays as described previously (**Figure 1**) (23, 28). Briefly, upon transfection of the LINE-1 reporter construct, a GFP reporter gene, which is interrupted by an intron, is only expressed after successful mRNA splicing, reverse transcription and integration into the host genome. Thus, GFP expression is used as a surrogate to quantify successful retrotransposition (**Figure S1**). First, we transfected HEK293T cells (293T) with LINE-1-GFP together with the different alleles of TRIM5α, the inactive control TRIM5α R437C, or empty vector and analyzed retrotransposition five days posttransfection by flow cytometry (**Figure 1A**). While cotransfection of TRIM5α R437C did not affect the frequency of LINE-1 retrotransposition as described previously (23), all of the tested TRIM5α alleles strongly inhibited LINE-1 activity (**Figure 1A**). Interestingly, one allele, TRIM5α H43Y, restricted LINE-1 even more efficiently than WT protein (**Figure 1A**). Active TRIM5α forms dimers and multimers, which is known to be crucial for retroviral restriction as well as for LINE-1 inhibition (23, 33). To prevent the formation of heterodimers between endogenous WT protein and exogenous TRIM5α H43Y, we generated 293T cells stably expressing shRNA targeting TRIM5α (293T-shTRIM5α) and transiently reconstituted the cells with the shRNA-resistant TRIM5α variants WT, H43Y, or R437C (WT^R^, H43Y^R^, R437C^R^) (**Figure 1B**). Also in these cells, we found that TRIM5α H43Y counteracts LINE-1 retrotransposition more efficiently than WT and that H43Y is active at lower concentrations compared to WT protein (**Figures 1B**; **S2**). Of note, we used the 293T-shTRIM5α cells and the shTRIM5α-resistant TRIM5α variants for all following assays to minimize the potential interference of the endogenous WT alleles with cotransfected TRIM5α H43Y. Next, we assessed the effect of TRIM5α H43Y on LINE-1-GFP integration events by digital droplet PCR (ddPCR) (23). Therefore, we transfected 293T-shTRIM5α cells with LINE-1-GFP construct together with TRIM5α WT or H43Y or empty vector. Five days posttransfection, we used FAM-labelled oligos specifically targeting the spliced GFP reporter gene as surrogate for successful integration into the host genome. In H43Y expressing cells, we found that LINE-1-GFP integrates were reduced by ~ 3-fold compared to WT expressing cells (**Figure 1C**). This finding confirms the enhanced inhibitory effect of the H43Y allele on LINE-1 as seen in the FACS-based assays and excludes any unspecific effects of H43Y on GFP reporter gene expression.

### TRIM5α H43Y is less active against exogenous retroviruses *in vitro*

**Figure 2:**
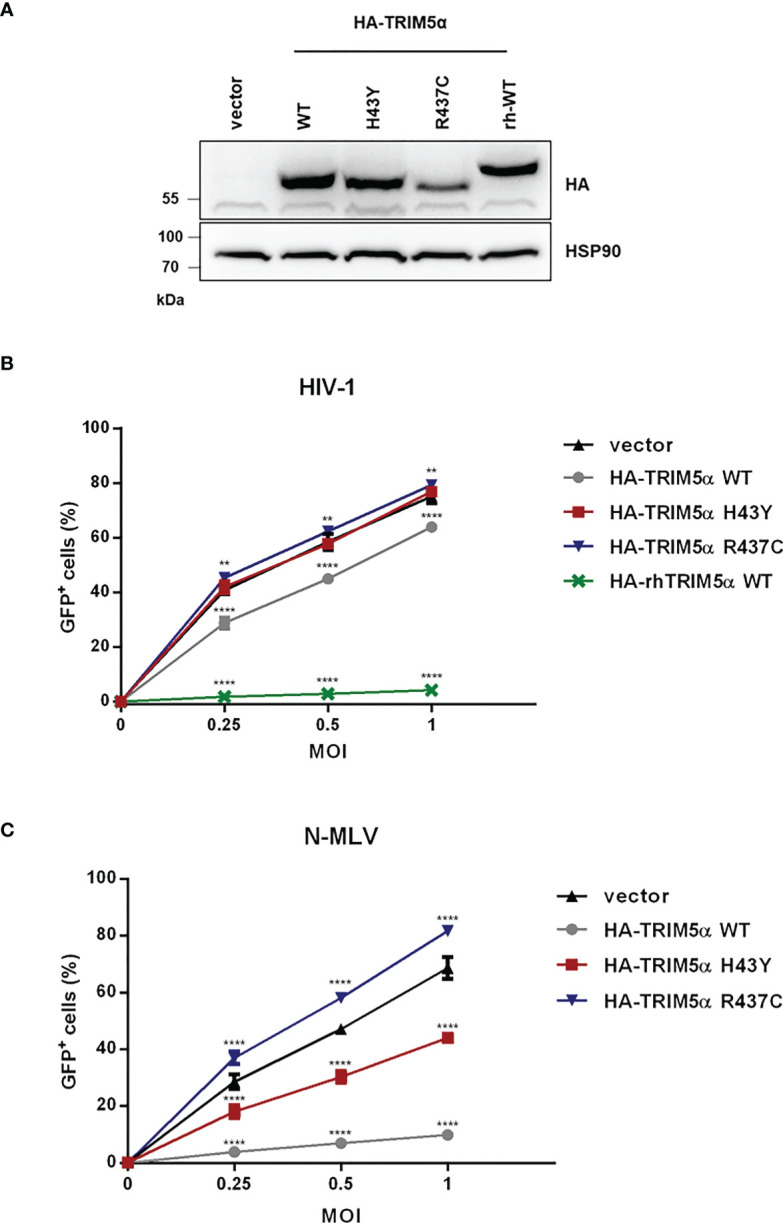
TRIM5α H43Y is less active against exogenous retroviruses *in vitro.* **(A)** CRFK cells stably expressing empty vector or HA-tagged human or rhesus (rh) TRIM5α variants were generated. Expression of the different HA-TRIM5a constructs was analyzed by immunoblot. Empty vector cells or CRFK cells stably expressing the indicated TRIM5α proteins were challenged with **(B)** VSV-G pseudotyped HIV-1-GFP reporter virus or **(C)** N-tropic Murine Leukemia Virus encoding GFP (N-MLV-GFP) at the indicated multiplicities of infection (MOI). Three days postinfection (dpi), the percentage of GFP-positive cells were quantified by flow cytometry and is shown as mean of triplicate infections with error bars indicating SD. One out of three independent experiments is shown. Statistical analysis were done using two-way ANOVA followed by Bonferroni’s multiple comparison test. **P<0.01, ****P<0.0001.

Since TRIM5α H43Y is highly active against LINE-1 retroelements but has originally been described as an impaired allele in the context of retroviral restriction (14), we next compared the activity of H43Y and TRIM5α WT against exogenous retroviral infection. Therefore, we transduced feline CRFK cells, which lack endogenous TRIM5α, with a retroviral vector to stably express either HA-tagged WT, H43Y, R437C or rhesus TRIM5α (rhTRIM5α) (**Figure 2A**). To assess the impact of the different variants on HIV-1 infectivity, we challenged the cells with VSV-G pseudotyped NL43-GFP reporter virus and quantified the number of infected cells by flow cytometry three days postinfection (dpi). As described before, we found that rhTRIM5α completely blocks infection, while human TRIM5α WT was only weakly active against HIV-1. Similar to LINE-1 inhibition, the minor antiviral effect of human TRIM5α on HIV-1 was abrogated by introducing the mutation R437C. However, in stark contrast to LINE-1 restriction, TRIM5α H43Y completely lost its anti-HIV-1 activity, similar to the negative control R437C (**Figure 2B**). In contrast to HIV-1, human TRIM5α is highly active against N-tropic Murine Leukemia Virus (N-MLV) (7, 8). To better discriminate between WT and H43Y in retroviral restriction, we infected TRIM5α expressing CRFK cells with VSV-G pseudotyped N-MLV-GFP (**Figure 2C**). As expected, TRIM5α WT efficiently inhibited N-MLV infection, while cells expressing the inactive variant R437C variant were permissive for infection. However, in line with our results on HIV-1, TRIM5α H43Y did not block N-MLV infection in CRFK cells. Together, we found that the human TRIM5α allele H43Y, which shows a strongly reduced activity against exogenous retroviruses, restricts endogenous LINE-1 retroelements more efficiently and more potently than the WT protein.

### Tyrosine at position 43 within the RING domain stabilizes TRIM5α structure

**Figure 3:**
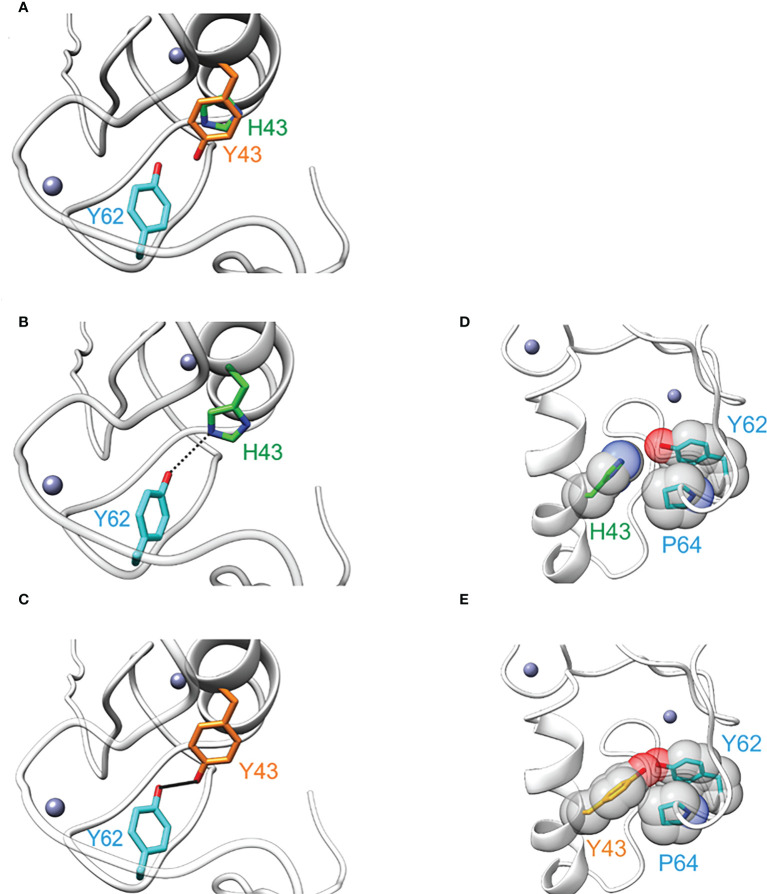
The amino acid exchange H43Y stabilizes the TRIM5α structure. **(A)** Overlay of TRIM5α WT (green) and the H43Y variant (orange) illustrates the differences in side chain length. **(B)** TRIM5α H43: the H43-Y62 distance allows for only weak hydrogen bond formation (represented by dashed line). **(C)** TRIM5α H43Y: the distance Y43-Y62 is shorter resulting in a stronger hydrogen bond (represented by a bold black line). **(D)** TRIM5α H43: the WT histidine shows no van der Waals contacts to P64. **(E)** TRIM5α H43Y: Y43 forms van der Waals contacts to P64 that stabilize the structure.

To assess the structural effect of the H43Y mutation, we performed energetic analyses based on the NMR-Ensemble of TRIM5α (PDB: 2ECV). The free energy change upon mutation to tyrosine was calculated for each of the 20 NMR-conformers, assuming either a protonated or a deprotonated form of histidine. Regardless of the analyzed structure and histidine protonation state, the exchange to tyrosine lead to a significant stabilization of the protein structure (**Figure 3**). A detailed analysis revealed that the two main contributions are a stronger hydrogen bond to Y62 (**Figures 3B, C**) and an improved Van der Waals packing with adjacent residues alongside with a better solvation energy (**Figures 3D, E**). In summary, the Y43 variant seems to be energetically more favorable leading to a structural stabilization of TRIM5α.

### TRIM5α H43Y potently activates immune signaling upon LINE-1 sensing

**Figure 4:**
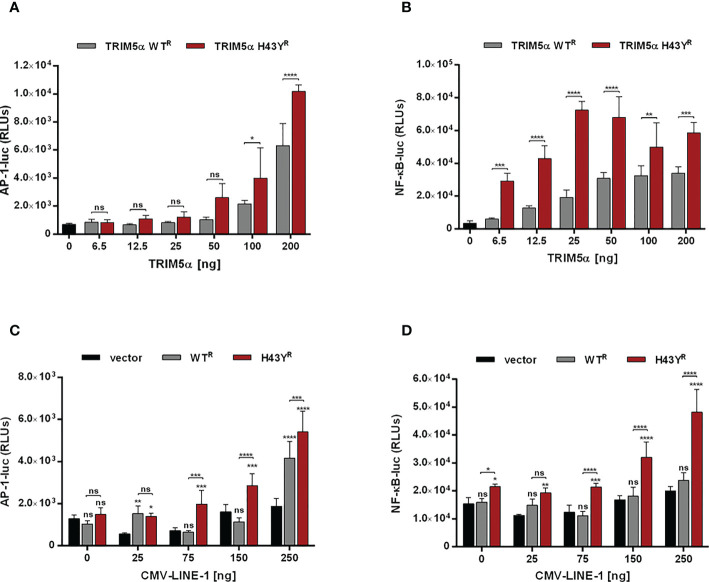
TRIM5α H43Y potently activates immune signaling cascades upon LINE-1 sensing. **(A, B)** 293T-shTRIM5α cells were transfected with reporter constructs expressing luciferase under the control of an **(A)** AP-1 or **(B)** NF-κB-dependent promoter and increasing amounts of TRIM5α WT or H43Y. **(C, D)** 293T-shTRIM5α cells were transfected with **(C)** AP-1 and **(D)** NF-κB reporter plasmids together with **(C)** 25 ng or **(D)** 6.5 ng of TRIM5α WT or H43Y and increasing amounts of a CMV promoter-driven LINE-1 expression construct (CMV-LINE-1). Two days posttransfection, cells were lysed and signaling induction was analyzed *via* luciferase assay. Relative luminescence units (RLUs) are shown as mean of quadruplicate transfections with error bars indicating SD. One out of three independent experiments is shown. Statistical analysis were done using two-way ANOVA followed by **(A, B)** Bonferroni’s multiple comparison test or **(C, D)** Tukey’s multiple comparison test. * P<0.1, ** P<0.01, *** P<0.001, **** P<0.0001, ns, not significant. ^R^ shRNA-resistant TRIM5α WT or H43Y.

The amino-acid exchange H43Y is located within the RING domain, which encodes the E3-ubiquitin ligase activity of TRIM5α. Since activation of immune signaling pathways by TRIM5α requires the formation of K63-ubiquitin chains (4), we asked whether the amino-acid exchange in the RING domain of TRIM5α might affect innate immune signaling. Therefore, we transfected 293T-shTRIM5α cells with luciferase reporter constructs under the control of an AP-1- or a NF-κB-dependent promoter together with increasing amounts of TRIM5α and quantified TRIM5α signaling activity by luciferase assay 48 h posttransfection (**Figures 4A, B**). Overexpression of exogenous TRIM5α has previously shown to be sufficient to trigger AP-1 and NF-κB pathways, independently of retroviruses (3). Similarly to WT protein, we observed that transient expression of TRIM5α H43Y also induces both AP-1 and NF-κB signaling pathways in a dose-dependent manner. Importantly, we found H43Y to be active already at lower concentrations and to induce NF-κB- and AP-1-dependent promoters to higher levels than WT protein (**Figures 4A, B**). Of note, comparing H43Y-mediated signaling with overexpression of the constitutively-active AP-1 and NF-κB activators MEK1-DD and IKK2-EE revealed that neither WT nor H43Y are activating the signaling pathways to full extent in our assays, thereby minimizing possible artificial effects due to overexpression (**Figure S3**). We also observed enhanced AP-1 and NF-κB activity upon transfection of H43Y in 293T cells, confirming that endogenous TRIM5α expression does not negatively affect the enhanced TRIM5a H43Y-mediated signaling (**Figures S4A, B**). Next, we asked whether the induction of AP-1 and NF-κB signaling in the presence of the “molecular pattern” LINE-1 RNP differs between the WT receptor TRIM5α and the H43Y allele. Thus, we cotransfected 293T-shTRIM5α cells with low amounts of TRIM5α expression plasmids, which are not sufficient to “auto-activate” signaling (**Figures 4A, B**), together with the luciferase reporter plasmids and increasing amounts of a plasmid encoding LINE-1 under control of a CMV promoter (CMV-LINE-1), the expression of which is unaffected by TRIM5α-mediated signaling. Importantly, we found that TRIM5α H43Y activated both AP-1 and NF-κB signaling at lower concentrations than WT protein in response to LINE-1 elements, suggesting a more efficient detection of LINE-1 in the cell (**Figures 4C, D**). Similarly, upon transfection of 293T cells, we found enhanced AP-1 and NF-κB signaling in the presence of TRIM5α H43Y and CMV-LINE-1, confirming the higher sensitivity of the receptor variant H43Y towards LINE-1 replication (**Figures S4C, D**).

### LINE-1 promoter activity is strongly reduced by TRIM5α H43Y

**Figure 5:**
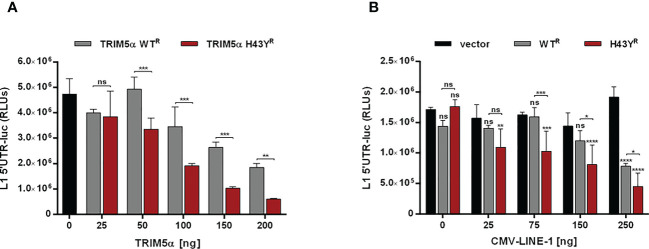
LINE-1 promoter activity is strongly repressed by TRIM5α H43Y. 293T-shTRIM5α cells were transfected with a LINE-1 promoter-driven luciferase reporter plasmid (L1 5’UTR-luc) together with **(A)** increasing amounts of TRIM5α WT or H43Y, or **(B)** 25 ng of TRIM5α WT or H43Y and increasing amounts of CMV-LINE-1. Two days posttransfection, cells were lysed and LINE-1 promoter activity was determined by luciferase assay. Relative luminescence units (RLUs) are shown as mean of quadruplicate transfections with error bars indicating SD. Statistical analysis were done using two-way ANOVA followed by **(A)** Bonferroni’s multiple comparison test or **(B)** Tukey’s multiple comparison test, *P<0.1, **P<0.01, ***P<0,001 ****P<0.0001, ns, not significant. One out of three independent experiments is shown. ^R^ shRNA-resistant TRIM5α WT or H43Y.

Previously, we showed that TRIM5α inhibits LINE-1 promoter activity through initiating AP-1 and NF-κB signaling pathways. Since H43Y activates AP-1 and NF-κB pathways more efficiently, we next asked whether H43Y also promotes a stronger downregulation of the LINE-1 promoter compared to the WT protein. We transfected 293T-shTRIM5α cells with a LINE-1 promoter luciferase reporter construct (L1 5’UTR-luc) together with increasing amounts of either TRIM5α WT or H43Y. Confirming our previous findings, TRIM5α WT suppressed LINE-1 promoter activity in a dose-dependent manner. The highly active variant H43Y, however, downregulated LINE-1 promoter-dependent transcription even stronger and already at lower doses compared to WT (**Figures 5A**; **S5A**). Next, we assessed the impact of TRIM5α on the LINE-1 promoter upon co-transfection of increasing amounts of a plasmid encoding LINE-1 under the control of a CMV-promoter (**Figures 5B**; **S5B**). Here, H43Y repressed LINE-1 promoter activity already in the presence of low amounts of CMV-LINE-1 and to a lower level compared to TRIM5α WT, suggesting that H43Y is sensing cytoplasmic LINE-1 RNPs more efficiently than the WT allele.

### Inhibition of TRIM5α H43Y-mediated immune signaling rescues LINE-1 promoter activity

**Figure 6:**
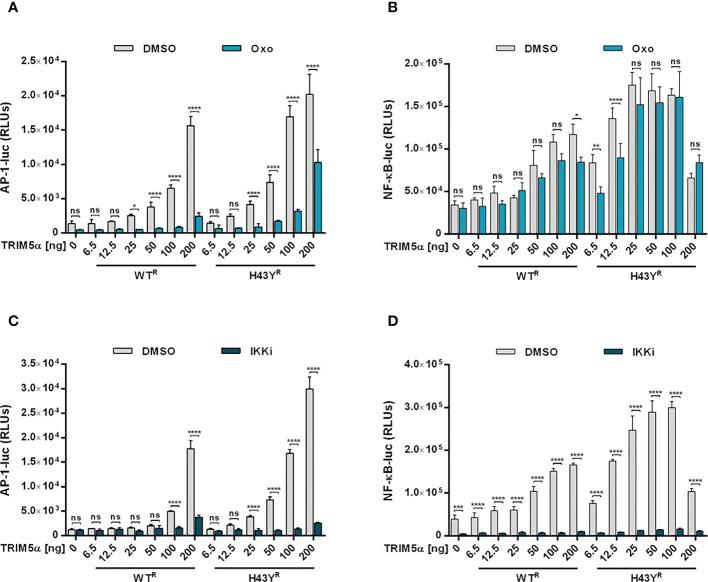
TAK-1 and NF-κB inhibitors block TRIM5α H43Y-mediated immune signaling. 293T-shTRIM5α cells were transfected with AP-1 **(A, C)** or NF-κB **(B, D)** luciferase reporter plasmids and increasing amounts of TRIM5α WT or H43Y. After 6 h, cells were treated with **(A, B)** 300 nM of 5Z-7-oxozeaenol (Oxo) or **(C, D)** a combination of 1 µM IKK inhibitor VII and 5 µM IKK inhibitor XII (IKKi). After two days, luciferase activity was analyzed. Relative luminescence units (RLUs) are shown as mean of quadruplicate transfections with error bars indicating SD. Statistical analysis were done using two-way ANOVA followed by Bonferroni’s multiple comparison test, *P<0.1, **P<0.01, ***P<0.001, ****P<0.0001, ns, not significant. One out of three independent experiments is shown. ^R^ shRNA-resistant TRIM5α WT or H43Y.

**Figure 7:**
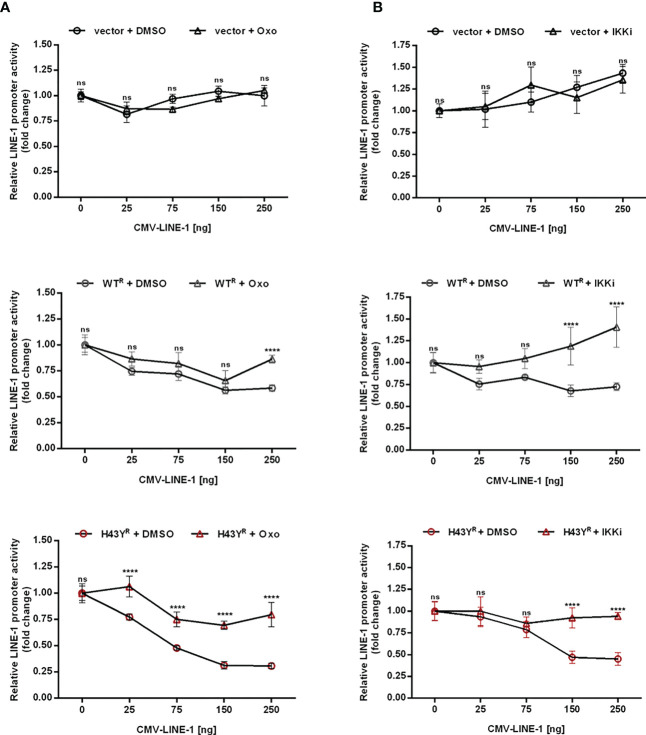
Inhibition of TRIM5α H43Y-mediated immune signaling rescues LINE-1 promoter activity. 293T-shTRIM5α cells were transfected with the LINE-1 promoter reporter plasmid (L1 5’UTR-luc) together with 25 ng of either an empty vector (upper panel), TRIM5α WT (middle panel), or H43Y (lower panel) and increasing amounts of a CMV-promoter-driven LINE-1 expression construct (CMV-LINE-1). At 6 h posttransfection, cells were treated with **(A)** 300 nM of Oxo or **(B)** a combination of IKK inhibitors (IKKi). After two days, LINE-1 promoter activity was assessed by luciferase assay. Luciferase activity is normalized on the condition lacking CMV-LINE-1 (0 ng) and displayed as mean of quadruplicate transfections with error bars indicating SD. Statistical analysis was done using two-way ANOVA followed by Bonferroni’s multiple comparison test, **** P<0.0001, ns, not significant. One out of three independent experiments is shown. ^R^ shRNA-resistant TRIM5α WT or H43Y.

Upon encounter of retroviral cores or LINE-1 RNPs, the ubiquitin ligase activity of TRIM5α initiates downstream AP-1 and NF-κB signaling cascades *via* activation of the kinase TAK-1. Since the H43Y variant induces these signaling pathways more efficiently, we next tested whether this allele also signals via TAK-1. First, we analyzed the effect of the TAK-1 inhibitor 5Z-7-Oxozeaenol (Oxo) on TRIM5α-mediated AP-1 and NF-κB signaling by luciferase reporter assay (**Figures 6A, B**, **S6A, B**, **S7**). While Oxo strongly reduced the levels of AP-1 signaling triggered by both WT and H43Y (**Figure 6A**) it barely had any effect on TRIM5α-mediated NF-κB signaling (**Figure 6B**). Interestingly, we found that at high levels of transfected plasmid, H43Y overcomes the Oxo-mediated block to AP-1 induction, suggesting that additional pathways of AP-1 activation exist (**Figure 6A**). Of note,it is well established that a crosstalk between the AP-1 and NF-κB signaling pathways exist at different levels and that NF-κB can control down-stream AP-1 activation (34, 35). Since we found that the Oxo-mediated block to TAK-1 doesn’t inhibit NF-κB signaling initiated by TRIM5α H43Y (**Figure 6B**), it is conceivable that the TAK-1 independent activation of NF-κB stimulates AP-1 signaling at high H43Y concentrations (**Figure 6A**). To analyze the role of NF-κB in H43Y signaling, we used a combination of IKK inhibitors (IKKi) in our signaling reporter assays. We found the TRIM5α WT and H43Y-mediated induction of NF-κB to be completely abrogated in presence of the inhibitors (**Figures 6D**,**S6C, D**, **S7**), while AP-1 signaling was only slightly affected in the presence of high amounts of TRIM5α (**Figure 6C**). To validate the inhibitory effect of TRIM5α-mediated immune signaling on LINE-1 promoter activity, we next evaluated the effect of the inhibitors on L1 5’UTR-luc in the presence of TRIM5α and LINE-1 RNPs. Therefore, we expressed the LINE-1 promoter reporter plasmid together with empty vector, TRIM5α WT or H43Y and increasing amounts of CMV-LINE-1 in the presence of Oxo (**Figure 7A**) or IKKi (**Figure 7B**). In empty vector transfected cells, neither IKKi nor Oxo affected LINE-1 promoter activity compared to DMSO treated cells (**Figures 7A, B**). In contrast, in TRIM5α WT and H43Y-transfected cells, the decrease in LINE-1 promoter activity, which correlated with the amount of cotransfected CMV-LINE-1, was rescued by Oxo and IKKi, indicating the importance of both AP-1 and NF-κB signaling pathways for H43Y-mediated inhibition of the LINE-1 promoter. Of note, similar to H43Y overexpression in signaling reporter assays (**Figure 6**), we found that the H43Y-mediated block to the LINE-1 promoter could not be fully rescued by Oxo at higher concentrations of CMV-LINE-1, supporting a role of the TAK1-independent NF-κB activation by TRIM5α H43Y in LINE-1 restriction (**Figure 7A**). Finally, we observed that both TAK-1 and IKK inhibitors relieved the block to LINE-1-GFP retrotransposition mediated by TRIM5α H43Y (**Figure S8**), confirming the essential role of both signaling pathways for the enhanced activity of TRIM5α H43Y against endogenous LINE-1 elements.

## Discussion

Within this study, we found that the frequent nonsynonymous SNP in human TRIM5α, H43Y, lost its antiviral activity but is highly active against endogenous LINE-1 elements. Upon encounter with its “immunogenic pattern”, LINE-1 RNPs, the H43Y variant of the receptor TRIM5α activates both NF-κB and AP-1 signaling pathways more potently than WT protein, resulting in a more efficient LINE-1 promoter repression. Our structural modeling of H43Y suggests a stabilized RING domain compared to TRIM5α WT. This finding might explain the more sensitive and efficient induction of the innate signaling pathways by H43Y, which depends on its ubiquitin ligase activity. Our results therefore suggest that the efficient inactivation of endogenous LINE-1 elements might be the evolutionary advantage that keeps the H43Y allele in the human population, despite the loss of a major part of its antiviral activity (**Figure 2**). This finding fuels the hypothesis that, from an evolutionary point of view, the “original” function of TRIM5α was not to restrict exogenous retroviruses but to contribute to genome stability by counteracting endogenous retroelements such as LINE-1. One hallmark of H43Y is the very efficient induction of innate immune signaling pathways (**Figure 4**). Especially for NF-κB signaling, we found an efficient and strong upregulation of the pathway already at low amounts of TRIM5a H43Y (**Figure 4B**). This suggests that the threshold for signal induction is lowered in H43Y compare to the WT allele, which might be a direct consequence of the stabilized conformation of the RING domain in H43Y as indicated by our structural analysis (**Figure 3**). In general, NF-κB expression and activity is highly regulated and cells employ a multilayered control system to keep NF-κB at bay. Since inappropriate NF-κB responses have been linked to autoimmune and inflammatory diseases (36), it will be interesting to determine next whether also the highly active SNP H43Y is associated with inflammatory diseases, which might represent an evolutionary disadvantage for the H43Y variant. On the other hand, however, TRIM5α-mediated signaling is thought to be controlled by a rapid turnover of the protein, although the degradation pathway employed remains unclear (4, 37). Since H43Y initiates these signaling pathways more effectively, it is possible that the enhanced signaling is also counteracted by a faster degradation of the protein, which would antagonize any detrimental effects of over-active NF-κB signaling.

Mechanistically, it is unclear why H43Y lost most of its antiviral activity despite being highly active against LINE-1. A role for TRIM5α as pattern recognition receptor for retroviral cores has been proposed earlier (3), resulting in NF-κB and AP-1 signaling upon interaction with and restriction of retroviral cores. Previously, we suggested a similar two step inhibitory mechanism for LINE-1 restriction by TRIM5α (23). At the same time, it will be interesting to determine why the otherwise highly active variant H43Y negatively affects retroviral restriction, especially since the interaction with the viral capsid protein is mediated by the SPRY domain of TRIM5α. Comparing the antiviral and the anti-LINE-1 activity of H43Y suggests that the strong activation of NF-κB and AP-1 by H43Y is more important for LINE-1 restriction than for the block to retroviral infection (**Figure 4**). Fletcher and colleagues recently generated a TRIM5α-Ubiquitin fusion protein to decipher the mechanism of TRIM5α-mediated signaling in great detail (4). Interestingly, this Ubiquitin-TRIM5α variant phenocopies TRIM5α H43Y activity and displays a loss of retroviral restriction while showing enhanced NF-κB signaling upon overexpression, thereby confirming our findings. One possible explanation for both phenotypes might lie within the prominent AP-1 and NF-κB binding sites present in retroviral promoter sequences. It has been shown that activation of both transcription factors boosts retroviral transcription, at least at the early stages of infection before antiviral effects of other AP-1 and NF-κB-stimulated genes kick in (38). In this case, the stronger activation of retroviral promoters upon H43Y-mediated signaling might outweigh the repressing effects of AP-1 and NF-κB-induced transcripts. In addition to retroviruses, TRIM5α has been shown to restrict the replication of Tick borne encephalitis virus (TBEV), a flavivirus transmitted by ticks (39). Upon entry of TBEV, TRIM5α has been reported to restrict viral replication by binding to the viral protease complex and inducing its degradation by mediating K48-linked ubiquitination. It will be interesting in the future to test whether the TRIM5α-induced AP-1 and NF-κB signaling also plays a role in TBEV restriction and whether H43Y is still able to restrict or, similar to retroviruses, has lost its antiviral activity against TBEV.

In conclusion, we identified a variant of the restriction factor and innate sensor TRIM5α, H43Y, which counteracts endogenous LINE-1 elements with high efficiency but lost its antiviral activity against exogenous retroviruses. Thus, our study suggests that the H43Y variant of TRIM5α persists within the human population due its enhanced protective effect against uncontrolled LINE-1 retrotransposition.

## Data availability statement

The original contributions presented in the study are included in the article/**Supplementary Material**. Further inquiries can be directed to the corresponding author.

## Author contributions

Study design: JL, BV, TG. Methodology: JL, SW, MC, HS, BV, TG. Investigation: JL, SW, MC, BV, HW. Data interpretation: JL, MC, HS, TG. Funding acquisition: TG, HS. Writing, review, editing: JL, MC, HS, TG. All authors contributed to the article and approved the submitted version.

## Supplementary material

Supplementary Figure 1 Illustration of the LINE-1-GFP construct used for retrotransposition assay. The LINE-1-GFP construct encodes a full-length LINE-1 sequence with a GFP cassette located in the 3’UTR in antisense orientation. The GFP cassette is expressed only after succesful splicing, reverse transcription and integration of LINE-1 into the host genome, and serves as a reporter for retrotransposition events.

Supplementary Figure 2 Low amounts of TRIM5α H43Y are sufficient to inhibit LINE-1 retrotransposition. 293T-shTRIM5α cells were transfected with LINE-1-GFP together with increasing amounts of TRIM5α WT, H43Y or R437C expressing vectors. Five days posttransfection, retrotransposition events were quantified by flow cytometry. Expression of the different shRNA-resistant TRIM5α proteins was confirmed by immunoblot using an HA-specific antibody. The percentage of GFP-positive cells is shown as mean of triplicate transfections. Error bars represent SD. Statistical analysis was done using two-way ANOVA followed by Tukey’s multiple comparison test, * P<0.1, **** P<0.0001, ns, not significant. ^R^ shRNA-resistant TRIM5α WT, H43Y, R437C.

Supplementary Figure 3 Activation of immune signaling by constitutively active MEK1-DD and IKK2-EE. (A) 293T-shTRIM5α cells were transfected with the luciferase reporter construct under the control AP-1-dependent promoter and increasing amounts of TRIM5α WT, H43Y or the constitutively active MEK1-DD. (B) 293T-shTRIM5α cells were transfected with the of NF-κB-luc construct together with increasing amounts of TRIM5α WT, H43Y or the constitutively active IKK2-EE. Two days posttransfection, cells were lysed and signaling induction was analyzed *via* luciferase assay. Relative luminescence units (RLUs) are shown as mean of quadruplicate transfections with error bars indicating SD. One out of two independent experiments is shown. Statistical analysis were done using two-way ANOVA followed by Tukey’s multiple comparison test. * P<0.1, ** P<0.01, *** P<0.001, **** P<0.0001, ns, not significant. ^R^ shRNA-resistant TRIM5α WT or H43Y.

Supplementary Figure 4 TRIM5α H43Y mediates enhanced signaling in 293T cells. 293T cells were transfected with AP-1- **(A, C)** or NF-κB-dependent **(B, D)** promoter reporter constructs and increasing amounts of TRIM5α WT and H43Y **(A, B)** or additional cotransfected with a CMV-LINE-1 construct **(C, D)**. Two days post-transfection, cells were lysed and signaling induction was analyzed *via* luciferase assay. Relative luminescence units (RLUs) are shown as mean of quadruplicate transfections with error bars indicating SD. One out of three experiments is shown. Statistical analysis were done using two-way ANOVA followed by Bonferroni’s multiple comparison test, * P<0.1, *** P<0.001, **** P<0.0001, ns, not significant.

Supplementary Figure 5 LINE-1 promoter activity is inhibited by TRIM5α H43Y in 293T cells. 293T cells were transfected with a LINE-1 promoter-driven luciferase reporter plasmid together with **(A)** increasing amounts of either TRIM5α WT or H43Y, or (B) 25ng of TRIM5α WT or H43Y and increasing amounts of CMV-LINE-1. Two days post-transfection, cells were lysed and L1 5’UTR-Luc activity was analyzed *via* luciferase assay. Relative luminescence units (RLUs) are shown as mean of quadruplicate transfections with error bars indicating SD. One out of three experiments is shown. Statistical analysis were done using two-way ANOVA followed by **(A)** Bonferroni’s correction or **(B)** Tukey’s *post hoc* test, * P<0.1, ** P<0.01, ***P<0,001 **** P<0.0001, ns, not significant.

Supplementary Figure 6 Innate signaling inhibition by the compounds 5Z-7-Oxozeaenol, IKKi VII and IKKi XII. 293T-shTRIM5α cells were transfected with either **(A, C)** AP-1-luc or **(B, D)** NF-κB-luc reporter constructs. 6 hours posttransfection, cells were treated with increasing concentrations of (A, B) 5Z-7-Oxozeaenol (Oxo) or **(C, D)** IKK inhibitors VII or XII. Two days later, cells were lysed and signaling inhibition were measured *via* luciferase assay. Relative luminescence units (RLUs) are shown as mean of quadruplicate transfections with error bars indicating SD. Statistical analysis were done using one-way ANOVA followed by Bonferroni’s correction. * P<0.1, ** P<0.01, *** P<0.001, **** P<0.0001, ns, not significant.

Supplementary Figure 7 The signaling inhibitors Oxo and IKKi do not affect cell viability. 293T-shTRIM5α cells were treated with either 300nM Oxo or a combination of 1µM IKKi VII and 5µM IKKi XII. Two days later, medium was removed and fresh medium containing the corresponding concentrations of signaling inhibitors was added for three additional days. After five days of treatment, cells were stained with the Fixable Viability Dye eFluor™ 780 and cell viability was analysed by flow cytometry. The viability of untreated cells (NT) was set at 100% and DMSO served as solvent control. Mean of triplicates treatments is shown with error bars representing SD. One out of two experiments is shown. Statistical analysis was done using one-way ANOVA followed by Bonferroni’s multiple comparison test, ns, not significant relative to NT.

Supplementary Figure 8 Blocking TRIM5α H43Y-mediated immune signaling cascades restores LINE-1 retrotransposition. 293T-shTRIM5α cells were transfected with the LINE-1-GFP plasmid together with either TRIM5α WT^R^ or H43Y^R^ and treated with Oxo or the combination of IKK inhibitors 6h and 48h post-transfection. Cells were harvested and GFP-positive cells were analyzed by flow cytometry five days post-transfection. Percentage of GFP+ cells was normalized for each group on the DMSO condition and displayed as fold change. Statistical analysis were done using two-way ANOVA followed by Tukey’s *post-hoc* test, * P<0.1, *** P<0.001 **** P<0.0001, ns, not significant. ^R^ shRNA-resistant TRIM5α WT or H43Y.
