# Supplementary figures and images for: A frequent SNP in TRIM5α strongly enhances the innate immune response against LINE-1 elements
Source: Front Immunol. 2023 Apr 26;14:1168589. doi: 10.3389/fimmu.2023.1168589 (PMC10169663; doi:10.3389/fimmu.2023.1168589)

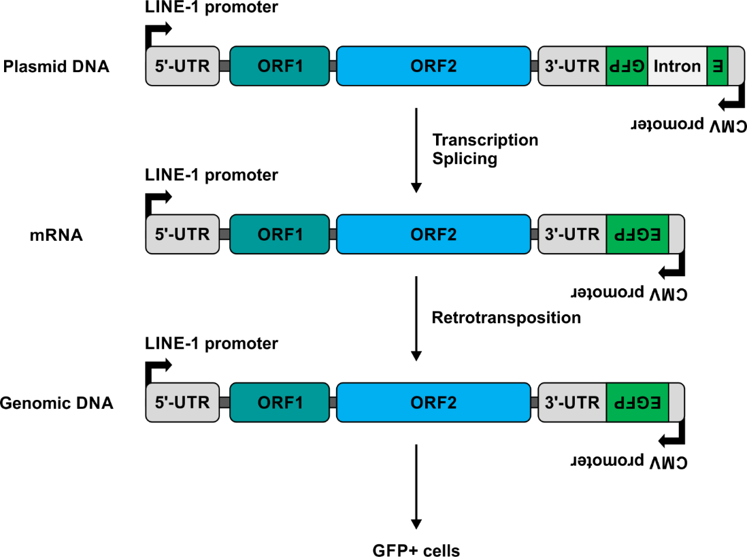

Supplement: Supplementary Figure 1 — Illustration of the LINE-1-GFP construct used for retrotransposition assay. The LINE-1-GFP construct encodes a full-length LINE-1 sequence with a GFP cassette located in the 3’UTR in antisense orientation. The GFP cassette is expressed only after succesful splicing, reverse transcription and integration of LINE-1 into the host genome, and serves as a reporter for retrotransposition events. [file Image_1.tif]

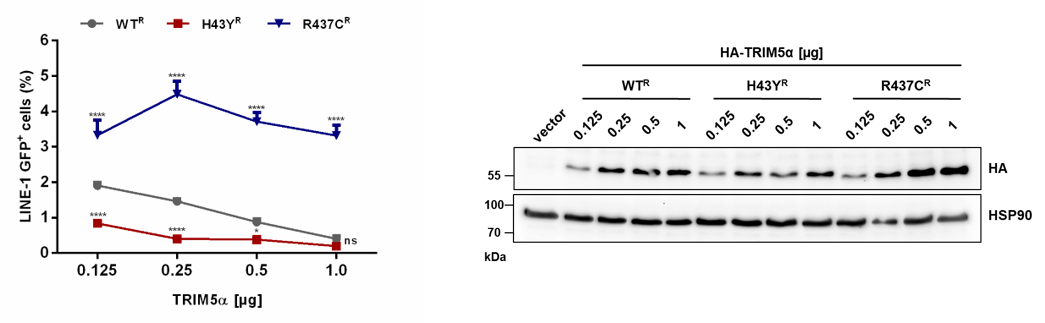

Supplement: Supplementary Figure 2 — Low amounts of TRIM5α H43Y are sufficient to inhibit LINE-1 retrotransposition. 293T-shTRIM5α cells were transfected with LINE-1-GFP together with increasing amounts of TRIM5α WT, H43Y or R437C expressing vectors. Five days posttransfection, retrotransposition events were quantified by flow cytometry. Expression of the different shRNA-resistant TRIM5α proteins was confirmed by immunoblot using an HA-specific antibody. The percentage of GFP-positive cells is shown as mean of triplicate transfections. Error bars represent SD. Statistical analysis was done using two-way ANOVA followed by Tukey’s multiple comparison test, * P<0.1, **** P<0.0001, ns, not significant. R shRNA-resistant TRIM5α WT, H43Y, R437C. [file Image_2.tif]

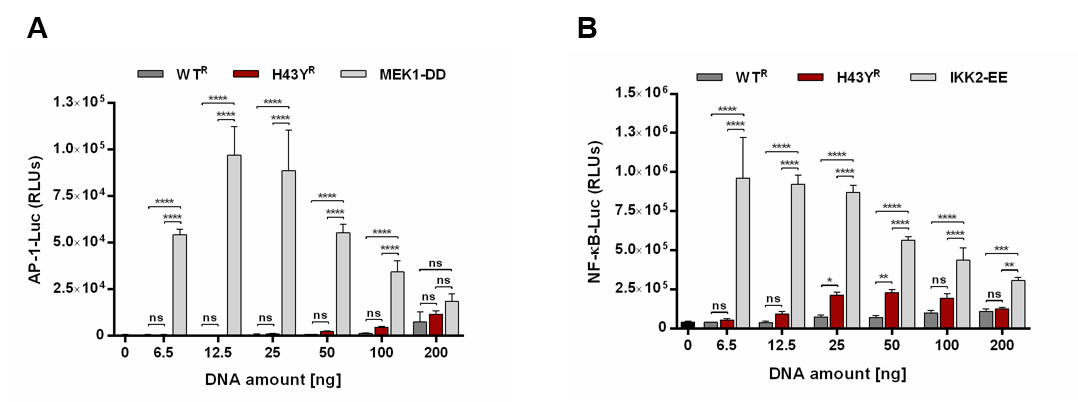

Supplement: Supplementary Figure 3 — Activation of immune signaling by constitutively active MEK1-DD and IKK2-EE. (A) 293T-shTRIM5α cells were transfected with the luciferase reporter construct under the control AP-1-dependent promoter and increasing amounts of TRIM5α WT, H43Y or the constitutively active MEK1-DD. (B) 293T-shTRIM5α cells were transfected with the of NF-κB-luc construct together with increasing amounts of TRIM5α WT, H43Y or the constitutively active IKK2-EE. Two days posttransfection, cells were lysed and signaling induction was analyzed via luciferase assay. Relative luminescence units (RLUs) are shown as mean of quadruplicate transfections with error bars indicating SD. One out of two independent experiments is shown. Statistical analysis were done using two-way ANOVA followed by Tukey’s multiple comparison test. * P<0.1, ** P<0.01, *** P<0.001, **** P<0.0001, ns, not significant. R shRNA-resistant TRIM5α WT or H43Y. [file Image_3.tif]

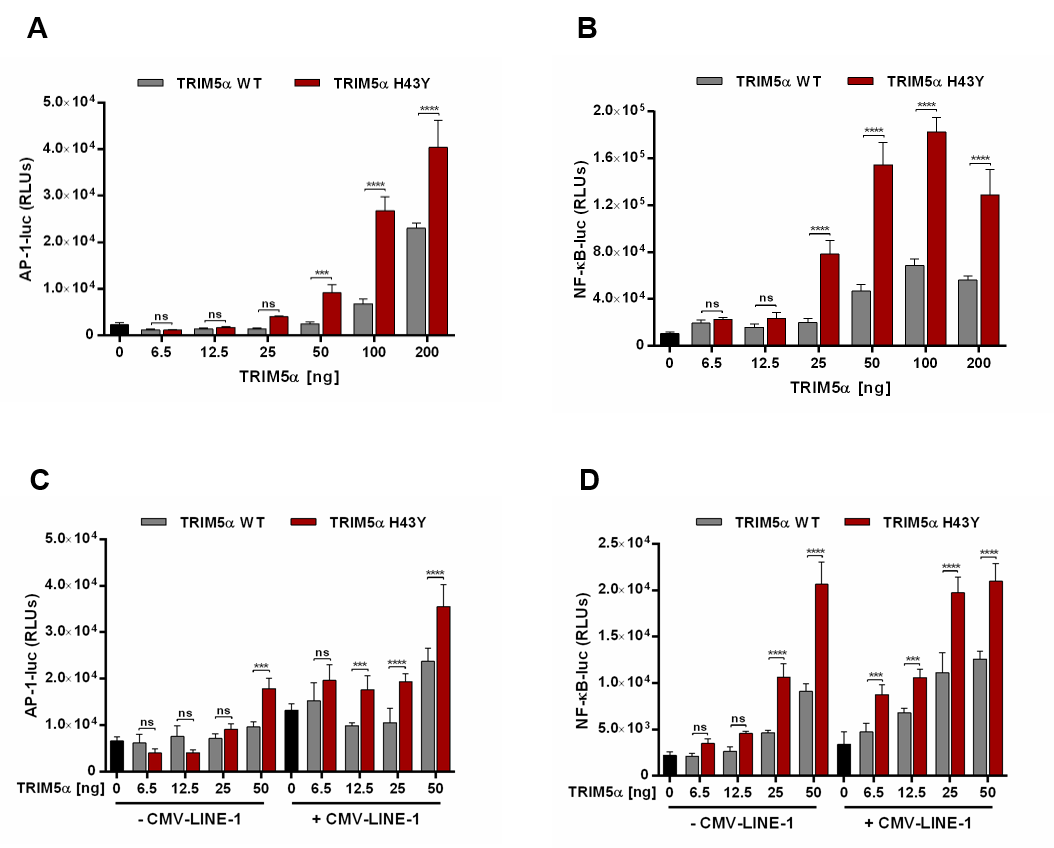

Supplement: Supplementary Figure 4 — TRIM5α H43Y mediates enhanced signaling in 293T cells. 293T cells were transfected with AP-1- (A, C) or NF-κB-dependent (B, D) promoter reporter constructs and increasing amounts of TRIM5α WT and H43Y (A, B) or additional cotransfected with a CMV-LINE-1 construct (C, D). Two days post-transfection, cells were lysed and signaling induction was analyzed via luciferase assay. Relative luminescence units (RLUs) are shown as mean of quadruplicate transfections with error bars indicating SD. One out of three experiments is shown. Statistical analysis were done using two-way ANOVA followed by Bonferroni’s multiple comparison test, * P<0.1, *** P<0.001, **** P<0.0001, ns, not significant. [file Image_4.tif]

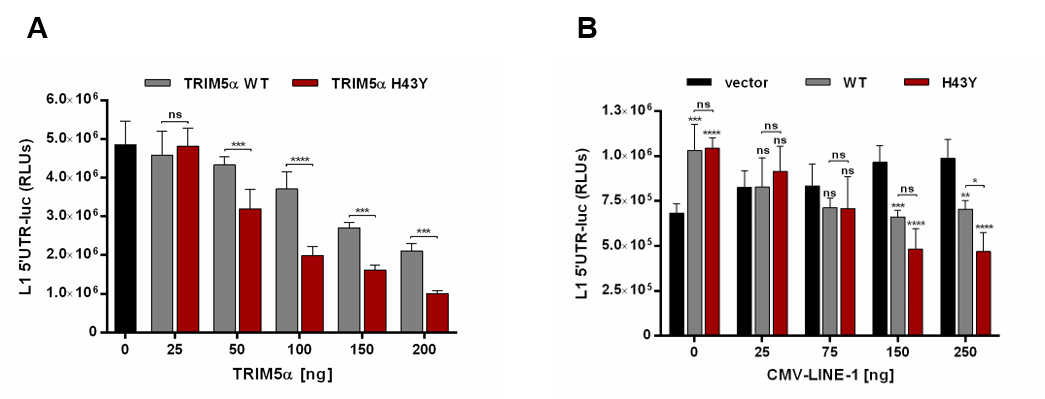

Supplement: Supplementary Figure 5 — LINE-1 promoter activity is inhibited by TRIM5α H43Y in 293T cells. 293T cells were transfected with a LINE-1 promoter-driven luciferase reporter plasmid together with (A) increasing amounts of either TRIM5α WT or H43Y, or (B) 25ng of TRIM5α WT or H43Y and increasing amounts of CMV-LINE-1. Two days post-transfection, cells were lysed and L1 5’UTR-Luc activity was analyzed via luciferase assay. Relative luminescence units (RLUs) are shown as mean of quadruplicate transfections with error bars indicating SD. One out of three experiments is shown. Statistical analysis were done using two-way ANOVA followed by (A) Bonferroni’s correction or (B) Tukey’s post hoc test, * P<0.1, ** P<0.01, ***P<0,001 **** P<0.0001, ns, not significant. [file Image_5.tif]

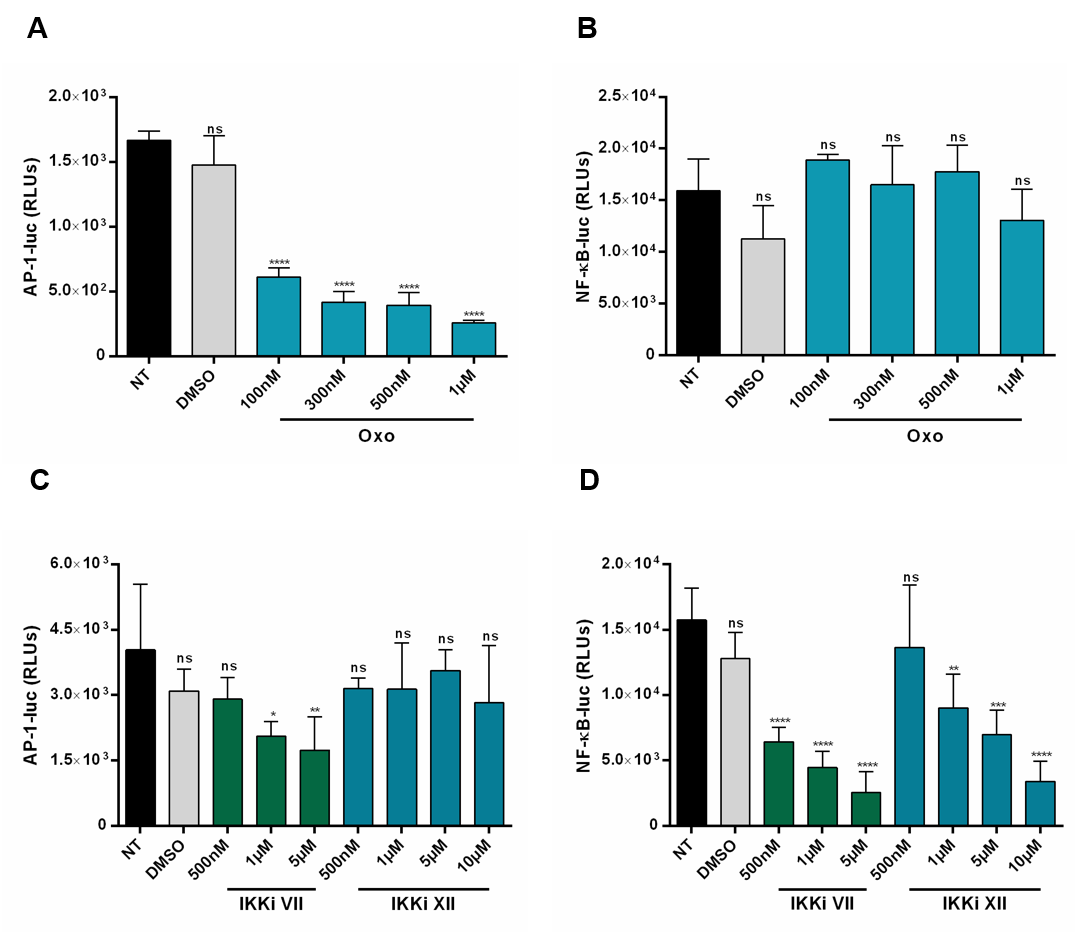

Supplement: Supplementary Figure 6 — Innate signaling inhibition by the compounds 5Z-7-Oxozeaenol, IKKi VII and IKKi XII. 293T-shTRIM5α cells were transfected with either (A, C) AP-1-luc or (B, D) NF-κB-luc reporter constructs. 6 hours posttransfection, cells were treated with increasing concentrations of (A, B) 5Z-7-Oxozeaenol (Oxo) or (C, D) IKK inhibitors VII or XII. Two days later, cells were lysed and signaling inhibition were measured via luciferase assay. Relative luminescence units (RLUs) are shown as mean of quadruplicate transfections with error bars indicating SD. Statistical analysis were done using one-way ANOVA followed by Bonferroni’s correction. * P<0.1, ** P<0.01, *** P<0.001, **** P<0.0001, ns, not significant. [file Image_6.tif]

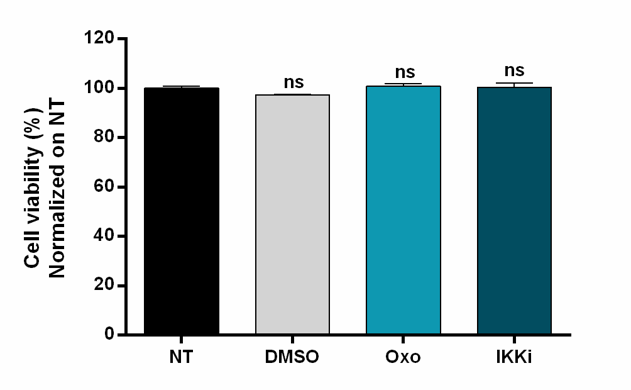

Supplement: Supplementary Figure 7 — The signaling inhibitors Oxo and IKKi do not affect cell viability. 293T-shTRIM5α cells were treated with either 300nM Oxo or a combination of 1µM IKKi VII and 5µM IKKi XII. Two days later, medium was removed and fresh medium containing the corresponding concentrations of signaling inhibitors was added for three additional days. After five days of treatment, cells were stained with the Fixable Viability Dye eFluor™ 780 and cell viability was analysed by flow cytometry. The viability of untreated cells (NT) was set at 100% and DMSO served as solvent control. Mean of triplicates treatments is shown with error bars representing SD. One out of two experiments is shown. Statistical analysis was done using one-way ANOVA followed by Bonferroni’s multiple comparison test, ns, not significant relative to NT. [file Image_7.tif]

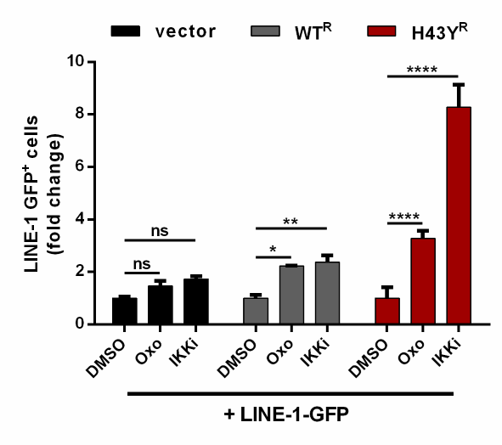

Supplement: Supplementary Figure 8 — Blocking TRIM5α H43Y-mediated immune signaling cascades restores LINE-1 retrotransposition. 293T-shTRIM5α cells were transfected with the LINE-1-GFP plasmid together with either TRIM5α WTR or H43YR and treated with Oxo or the combination of IKK inhibitors 6h and 48h post-transfection. Cells were harvested and GFP-positive cells were analyzed by flow cytometry five days post-transfection. Percentage of GFP+ cells was normalized for each group on the DMSO condition and displayed as fold change. Statistical analysis were done using two-way ANOVA followed by Tukey’s post-hoc test, * P<0.1, *** P<0.001 **** P<0.0001, ns, not significant. R shRNA-resistant TRIM5α WT or H43Y. [file Image_8.tif]
